# BlastFrost: fast querying of 100,000s of bacterial genomes in Bifrost graphs

**DOI:** 10.1186/s13059-020-02237-3

**Published:** 2021-01-11

**Authors:** Nina Luhmann, Guillaume Holley, Mark Achtman

**Affiliations:** 1 Warwick Medical School, University of Warwick, Coventry, UK; 2 Faculty of Industrial Engineering, Mechanical Engineering and Computer Science, University of Iceland, Reykjavík, Iceland

## Abstract

BlastFrost is a highly efficient method for querying 100,000s of genome assemblies, building on Bifrost, a dynamic data structure for compacted and colored de Bruijn graphs. BlastFrost queries a Bifrost data structure for sequences of interest and extracts local subgraphs, enabling the identification of the presence or absence of individual genes or single nucleotide sequence variants. We show two examples using *Salmonella* genomes: finding within minutes the presence of genes in the SPI-2 pathogenicity island in a collection of 926 genomes and identifying single nucleotide polymorphisms associated with fluoroquinolone resistance in three genes among 190,209 genomes. BlastFrost is available at https://github.com/nluhmann/BlastFrost/tree/master/data.

**Supplementary Information:**

The online version contains supplementary material available at (10.1186/s13059-020-02237-3).

## Introduction

Recent advances in DNA sequencing technologies have reduced sequencing costs and hands-on time, and whole-genome sequencing of bacterial pathogens is being routinely performed by public health organizations. The resulting sequence reads and genome assemblies are deposited in the public domain [1–3], enabling comparative analyses of 100,000s of genomes [4, 5] from individual bacterial genera for evolutionary or epidemiological investigations.

New sequencing data are now routinely uploaded to public databases such as the Sequence Read Archives (SRA [6]), which provide ready access to extensive collections of sequencing data for many bacterial genera. Sequences from specific bacterial pathogens are also available as curated collections of genomic assemblies bundled with their metadata together with dedicated tools for population genomic analyses. Such databases include for example PubMLST [7] and EnteroBase [3].

The analysis of genomic sequences by phylogenetic approaches can yield insights into evolutionary distances for 1000s of bacterial genomes. However, large comparative studies based on sequencing data are limited by computing resources and calculation speed [5]. Even the seemingly simple task of identifying all bacterial strains within a collection that contains a specific antimicrobial resistance gene or other genes of interest is a computational challenge for the large data sets that are currently available. The most popular methods for sequence comparison are BLAST [8] and its successors. However, these alignment-based methods do not scale well for queries of the presence or absence of genes in large data sets. As a result, as recently reviewed by Marchet et al. [9], the alignment step is replaced in some recent software by a *k*-mer approach [10], in which sets of short sub-sequences of fixed length *k* are compared between a query and a sequence database. These approaches were implemented because *k*-mers can directly identify diverse genetic modifications such as single nucleotide polymorphisms (SNPs), insertions or deletions from short read sequences, and do not require assembled genomes or an explicit reference genome.

One recent *k*-mer-based method, BIGSI, employs a data structure that stores a Bloom filter [11] of *k*-mers for each genome in a database, and can subsequently index and search very large databases of bacterial and viral sequences [12]. BIGSI queries are very efficient, but the European Nucleotide Archive (ENA) was already so large in 2016 that creating a BIGSI index took months. Furthermore, BIGSI was designed for dealing with genetically diverse collections of data, and other methods and different data structures might be more efficient for creating a query index of sequence data from closely related genomes. A potentially faster approach for the construction of indices would be to index sets of *k*-mers in a de Bruijn graph [13], where shared *k*-mers are automatically collapsed into single nodes. Collapsing *k*-mers that are shared between closely related genomes would decrease both the storage space for the index and the search space for subsequent queries. Recent implementations of such an approach include Mantis [14], Rainbowfish [15], and VARI-Merge [16]. They build joint de Bruijn graphs for multiple genomes, coloring nodes by their source genomes (colored de Bruijn graphs [17]), and can traverse both the shared paths in the graph which represent conserved regions as well as diverging paths which represent variable regions. However, the implementations of these methods do not scale well enough to efficiently handle a modern, large sequence collection [18]. For example, VARI-Merge was benchmarked on a data set of 16,000 *Salmonella* genomes [16], but the *Salmonella* database in EnteroBase already contains >250,000 genomes.

The recent development of Bifrost [18] introduced a memory efficient, dynamic data structure for indexing either colored or non-colored compacted de Bruijn graphs. It presents a broad range of functions that support querying both sequences and colors, annotating individual vertices, and editing Bifrost graphs while preserving their compaction. The implementation of Bifrost facilitates its ability to rapidly build joint graphs of 100,000s of genomes and permits incremental updates of these large graphs with additional data. However, Bifrost only implements basic *k*-mer querying. Here, we introduce BlastFrost, a method implemented in C++ for similarity searches in Bifrost graphs by rapid *k*-mer matching. BlastFrost uses the underlying Bifrost graph structure to extract subgraphs defined by a query, and can thereby efficiently extract sequence variants of the query from a data base of 100,000s of bacterial genomes. Here, we show that BlastFrost performs better than Blast and BIGSI with closely related genomes, and illustrate its features by case studies on the identification of genomic islands and of individual mutations in antimicrobial resistance genes.

## Results

Uncompacted de Bruijn graphs of genomic sequences are a popular graph data structure consisting of nodes representing sequences of *k*-mers within the input genomes. Edges in the graph represent fixed overlaps of length *k*−1 between neighboring nodes and can therefore be implicit. Bifrost [18] indexes bacterial genomes in a time and memory efficient implementation of a compacted and colored de Bruijn graph. Here, maximal paths of multiple sequential, non-branching nodes are compacted into single nodes (unitigs) by collapsing the overlaps. In addition, each node is assigned a set of colors representing all input genomes containing the corresponding *k*-mers of the unitig. We henceforth refer to this particular form of compacted and colored de Bruijn graphs as Bifrost graphs.

**Fig. 1:**
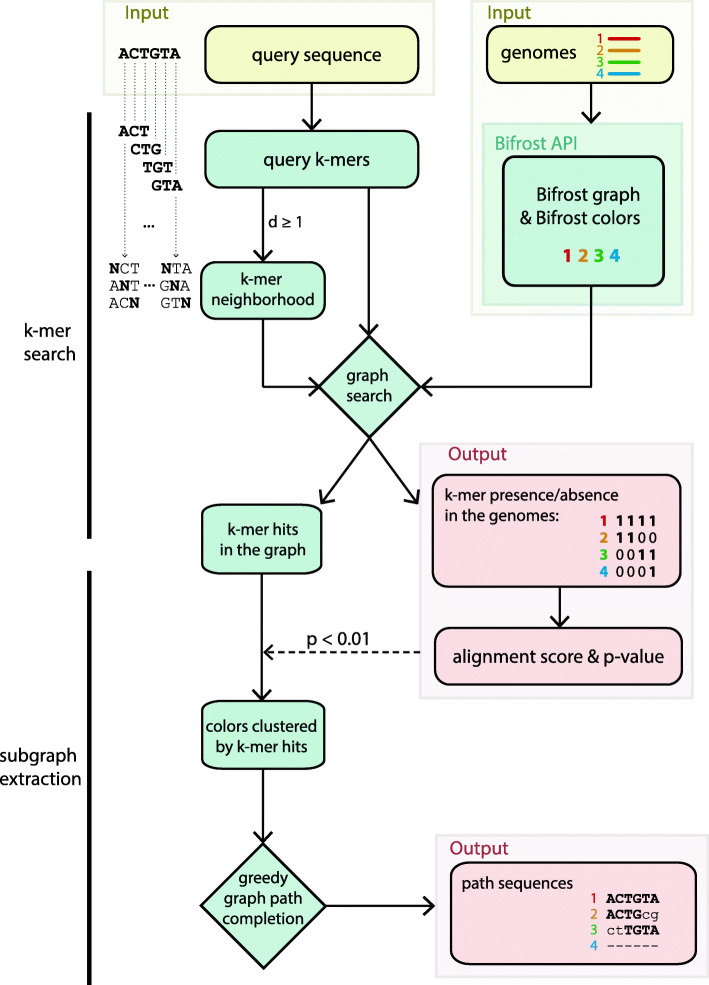
Overview of the flow and algorithms used by BlastFrost. BlastFrost is a command line program whose inputs consist of pre-computed files generated by Bifrost that specify the graph and colors plus a user-defined FASTA file of query sequences. BlastFrost searches a *k*-mer neighborhood for the parameters *k* (*k*-mer length) and *d* (Hamming distance) and estimates an alignment score and *p* value for each query sequence. The presence or absence of hits are recorded in a tab-delimited file containing the query ID, color ID, and binary presence/absence data. When run with the input parameter −*e*, BlastFrost uses these data to extract subgraphs, and appends their path sequences to the output file

BlastFrost relies on Bifrost graphs. As depicted in Fig. 1, we implemented a *k*-mer search function in BlastFrost which can identify the presence or absence of a query sequence in any of the genomes in a Bifrost graph. The results of that search can be used for subgraph extraction (Fig. 1 bottom) of query matches in order to identify all variants of the query sequence in the Bifrost graph. The following paragraphs provide an overview of the method. Algorithmic details can be found in Supplemental Material.

### BlastFrost query search

The input for BlastFrost consists of a Bifrost graph file in GFA format plus an index of the colors of each *k*-mer in each unitig, pre-computed for a certain *k* value. We henceforth refer to the genomes indexed in the graph as colors. The input parameters to BlastFrost also include a link to a FASTA file containing one or more query sequences.

For each query sequence, BlastFrost calculates a set of overlapping *k*-mers, using the same value of *k* that was used to build the Bifrost graph. This set is then used to search for the query sequence in all genomes in the graph, relying on specific functions from the Bifrost API that determine the presence of each *k*-mer and its colors in the Bifrost graph. Each query results in a binary sequence for each color of 1s and 0s representing *k*-mer hits and misses. The *k*-mer-based search in the graph explicitly assumes that overlapping *k*-mers of the same color are also contiguous in the underlying genome, which speeds up computation. BlastFrost speeds up computations even further by taking advantage of the fact that Bifrost graphs are compacted into unitigs which encode non-branching nodes as single nodes, and assumes that the color set of a unitig is the same as the individual color sets of each *k*-mer in that unitig.

A single nucleotide substitution between a query and a color will result in *k* mismatching *k*-mers, assuming that the size of *k* was large enough to avoid random hits in the genomes. The resulting binary sequence would then contain a stretch of *k* 0’s in the binary hit sequence for that query. Deletions are also characterized by runs of 0’s that are potentially smaller than *k*, while insertions and multiple substitutions can lead to longer runs of 0’s in the hit sequence. In order to evaluate the significance of *k*-mer hits between a query and a specific color, we adopted the BLAST approach for computing an *E* value [19] based on an estimated alignment score, derived from the lengths of 0 runs in the *k*-mer hits.

To increase the sensitivity of the *k*-mer-based query, BlastFrost allows additional querying of all *k*-mers related to a query *k*-mer by a Hamming distance smaller than or equal to an input parameter *d*. We refer to this set of additional *k*-mers as *k*-mer neighborhoods (Fig. 1). In the following evaluation, we present the necessity for this increased sensitivity, as well as some of the resulting trade-offs.

### BlastFrost subgraph extraction

The raw results on *k*-mer hits from a Bifrost graph are not immediately informative on the genomic locations of the query hits, the numbers of copies of those query sequences in each genome, or on syntenic relationships. For any specific query, each binary sequence of *k*-mer hits represents a potentially incomplete path of nodes for each color in the graph interrupted by nucleotide changes that were not included in the *k*-mers that are shared between the query and the genome. BlastFrost can account for these potential gaps by extending the *k*-mer hit results, and produce a subgraph for each successful *k*-mer query. Starting from the first unitig in the original *k*-mer hit list for a specific color, BlastFrost greedily completes a path by traversing non-branching paths of the same color within the graph, i.e., each unitig initially found in the *k*-mer search is tested for all successor unitigs with the same color. The subgraph is then used to reconstruct the corresponding sub-sequence of each color from the path in the Bifrost graph.

To avoid completing the same paths more than once, BlastFrost clusters colors sharing *k*-mer hits, completes all their paths simultaneously, and removes colors from those clusters that are absent in intervening unitigs. For each path and its accompanying colors, BlastFrost output the genome sequence in addition to the binary sequence mentioned above. These data allow ready identification of variant positions that distinguish the query from the extracted path sequences.

### Evaluation and benchmarks

#### Precision and sensitivity of identifying the presence or absence of all genes in a pan-genome

We used the whole-genome MLST (wgMLST) scheme for the genus *Salmonella* in EnteroBase [3, 20] to test the accuracy of BlastFrost for detecting sequence variants of a large number of query sequences in a large number of related genomes. That wgMLST scheme consists of 21,065 single copy orthologs which had been derived from a pan-genome of 537 representative genomes of *Salmonella* with PEPPAN [20, 21]. EnteroBase identifies diverse sequence variants of those loci in each assembled genome by combining BLASTN [8] nucleotide and UBLAST [22] amino acid queries, and also scores the absence of significant hits for each genome. Thus, this data set is ideal for testing the efficiency of the detection of presence and absence of multiple genes because both the presence and absence as well as the genome-specific sequence are known for all 21,065 loci in all *Salmonella* genomes in EnteroBase.

Bifrost created a graph of 926 representative *Salmonella* genomes from EnteroBase [20] in less than 24 min and required less than 5 GB of memory. The graph occupies 2.3 GB of disk space, and it contains more than 33 million unitigs.

We ran BlastFrost (parameter *d*=1 to support inexact searches) on this graph with 21,065 query sequences, consisting of one representative allele for each locus, and extracted all allelic variants from the corresponding subgraphs.

**Fig. 2:**
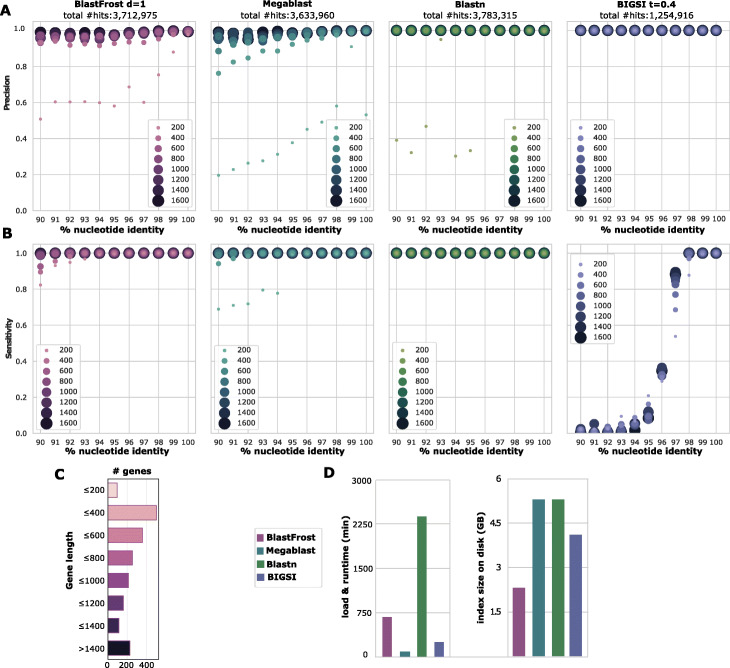
Comparisons of BlastFrost (subgraph extraction mode, *d* = 1), Megablast, Blastn, and BIGSI (cutoff *t* = 0.4) for 21,065 queries consisting of the reference alleles for all wgMLST loci in the EnteroBase *Salmonella* database against 926 representative *Salmonella* genomes [20]. **a** Precision: proportion of all query hits which corresponded to sequence variants (alleles) in EnteroBase with >90*%* sequence coverage. **b** Sensitivity: proportion of alleles in EnteroBase which were recovered by the respective methods. **c** Distribution of numbers of wgMLST loci by sequence length. **d** Runtime and disk storage space for all four methods

To calculate precision and sensitivity, we scored extracted sequences that covered at least 90% of the query sequence in a pairwise alignment as being correct. We bin query hits by the nucleotide identity between the query and the EnteroBase allele, or the nucleotide identity between the query and the search result if an allele is not stored in EnteroBase. We also performed similar analyses with the programs Megablast, which is the default version used by Blast [8], the classical version of BLASTN, and BIGSI [12] with the parameter *t*=0.4 to support inexact searches. Initial comparisons showed that the precision of all of these methods was very low for genes of less than 200-bp length (Fig. 2a), except for BIGSI which had extremely high precision, and the following description is restricted to genes that were larger than 200 bp. The precision with BlastFrost was at least 95% for alignments with at least 90% nucleotide identity. MegaBlast had somewhat lower precision for genes of less than 400 bp, whereas BLASTN retained very high precision at all levels. We also examined the nature of the false-positive hits by BlastFrost according to our criteria of correct hits to determine whether these were potentially truly absent in the corresponding genomes. Indeed, all false positives, including genes of <200 bp length, were found in those genomes with BLASTN, indicating that false positives may have been scored because EnteroBase scoring excludes repetitive DNA elements, including overlapping or duplicated sequences, whereas BlastFrost finds all sequences, including such repetitive DNA. Sensitivity with BlastFrost was 100% down to 94% nucleotide identity and then dropped to >94*%* at 90% identity (Fig. 2b). Megablast and BLASTN yielded almost perfect sensitivity throughout. BIGSI failed dramatically with nucleotide identities below 95%, because it only identified a limited number of hits in total despite a low cutoff parameter *t*, explaining its superb precision values. In summary, BlastFrost correctly identified all sequence variants down to 90% sequence identity with a gene length of >200 bp.

The runtime of Megablast and BIGSI was much faster than that of BlastFrost while the runtime of BLASTN was threefold slower (Fig. 2d). BlastFrost also required the least disk space for the genome indices of all these programs (Fig. 2e).

#### Benchmarking

The initial results in Fig. 2 indicate that BlastFrost was slower than Megablast and BIGSI. We therefore compared the time and memory requirements between these different methods and minimap2 [23], another widely used software tool, in greater detail to explore their relative strengths.

**Fig. 3:**
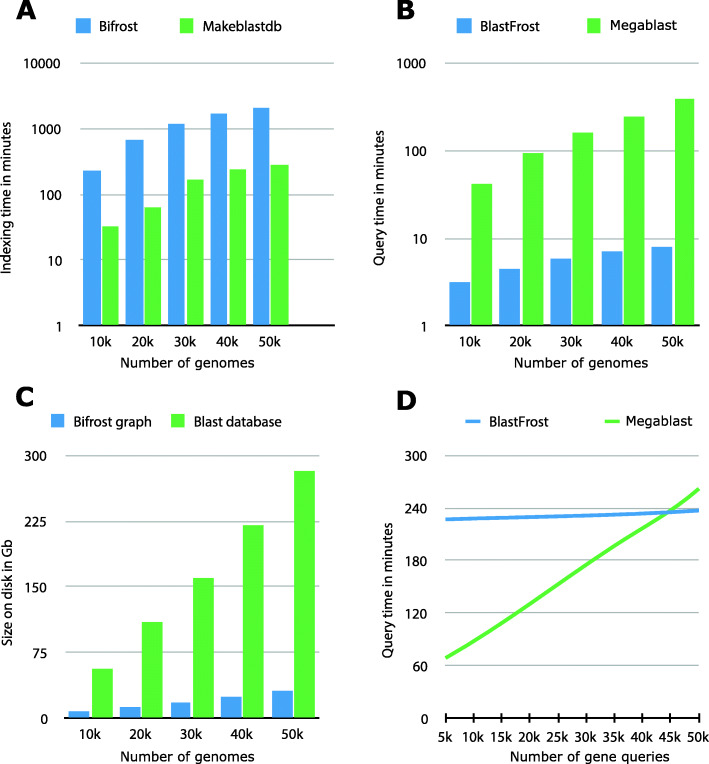
Comparison of runtime and space requirements for Bifrost, BlastFrost, and Blast with 10,000−50,000 randomly selected *Salmonella* genome assemblies. **a** Indexing time needed by Bifrost and makeblastdb (blast) on 10,000−50,000 randomly selected genome assemblies. **b** Query time for 6500 AMR genes against the previously indexed genome assemblies. **c** Disk space occupied by indexed assemblies. **d** Query time for 5000−50,000 genes against an index of 10,000 genome assemblies

We compared the times needed by BlastFrost and Bifrost to Megablast for indexing 10,000−50,000*Salmonella* genome assemblies and querying them with multiple individual genes (Fig. 3). The indexing step in BLAST is close to an order of magnitude faster than graph construction by Bifrost (Fig. 3a). However, after loading the graph, BlastFrost is more than tenfold faster than MegaBlast at searching the constructed graph for the presence of 6500 AMR genes (Fig. 3b) because of the extensive indexing information within the Bifrost graph. The Bifrost graph also needs much less disk space than a BLAST database (Fig. 3c). Megablast is clearly faster than BiFrost plus BlastFrost for a single phase of creating an index 10,000 genomes plus a single round of querying up to 50,000 genes (Fig. 3d). Under conditions where a genome database grows continuously and gene queries are conducted repeatedly, Bifrost plus BlastFrost would be much quicker because Bifrost can rapidly and continuously expand its pre-constructed graph to include additional genomes whereas BLAST needs to calculate a complete index for each additional genome.

**Fig. 4:**
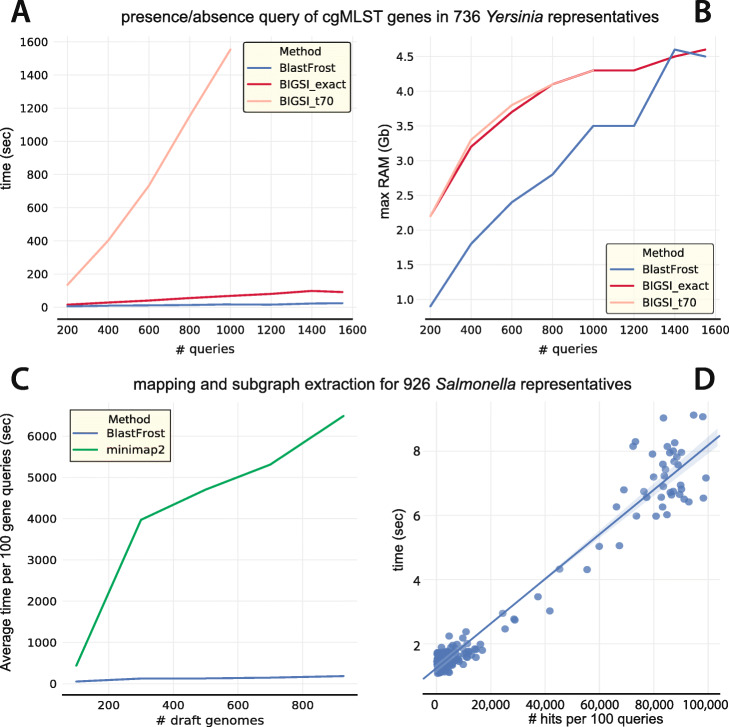
Comparison of BlastFrost to BIGSI (**a**, **b**) and minimap2 (**c**, **d**). **a**, **b** Runtime and memory required for querying 736 genomes of *Yersinia pestis* [3] as a function of numbers of queries for BlastFrost with *k*-mer neighborhood *d*=1 in comparison to both exact and inexact BIGSI queries. **c** Runtime for subsets of 926 *Salmonella* genomes [20] as a function of numbers of draft genomes for BlastFrost with *k*-mer neighborhood *d*=1 in comparison to minimap2. **d** Time required by BlastFrost to extract a subgraph by number of hits for a 100 gene query

We also investigated calling the presence or absence of core genes with BlastFrost and BIGSI [12] on a data set of 736 genomes from representative strains of *Yersinia pestis*, whose genomic diversity is very limited [3]. Creating indices of the 736 genomes was much faster with Bifrost than with BIGSI (Supplemental Material). The query time and maximum RAM usage were measured with BlastFrost and BIGSI for 200−1600 random core gene sequences. BlastFrost was timed in an inexact search for *k*-mer hits with up to one nucleotide mismatch (parameter *d*=1). For BIGSI, we timed an exact search function as well as an inexact search function for query hits containing at least 70% of the query sequence *k*-mers (parameter *t*=0.7). The (inexact) BlastFrost query yielded the same hits as the inexact BIGSI search, but BIGSI was much slower. BlastFrost searches were slightly faster than the exact BIGSI searches (Fig. 4a) and used much less RAM for less than 1200 queries.

Finally, we timed subgraph extraction compared between BlastFrost and minimap2 [23], which is the currently most efficient mapping tool for both short reads as well as chromosome-scale alignments. The average speed needed to extract 100 genes from the wgMLST *Salmonella* scheme described above from was measured across subsets of the 926 representative *Salmonella* genomes. The measurement showed BlastFrost is much faster than minimap2 (Fig. 4c). The time needed for BlastFrost to extract a subgraph is dependent on the number of hits for that query (Fig. 4d), but it achieves a slightly sub-linear growth in time requirement because identical genome segments can be found in multiple genomes within a bacterial genus.

### Applications

We took advantage of the large genomics databases available in EnteroBase to demonstrate the abilities of BlastFrost to find variably present genomic elements and to identify single nucleotide variants of individual genes. For genomic elements, we searched the 926 representative genomes of *Salmonella* for known genes in the SPI-2 *Salmonella* pathogenicity island [24–26]. For nucleotide variants, we screened the entire EnteroBase *Salmonella* database for specific substitutions in three genes that are associated with fluoroquinolone resistance in *Salmonella* [27].

#### Genomic islands

Genomic islands consist of clustered genes from the accessory genome that can be acquired by bacteria through horizontal gene transfer, or which are lost due to gene deletion [28, 29]. Pathogenicity islands are a distinct sub-class of genomic islands, which can range in size from 10 to 200 kb, and encode genes which can contribute to the virulence of the bacteria [26, 30]. SPI-2 is such an island which seems to have been acquired by *Salmonella* after the divergence of *S. bongori* and *S. enterica* from their common ancestor. Subsequently, *S. enterica* split into multiple so-called subspecies [20, 31].

**Fig. 5:**
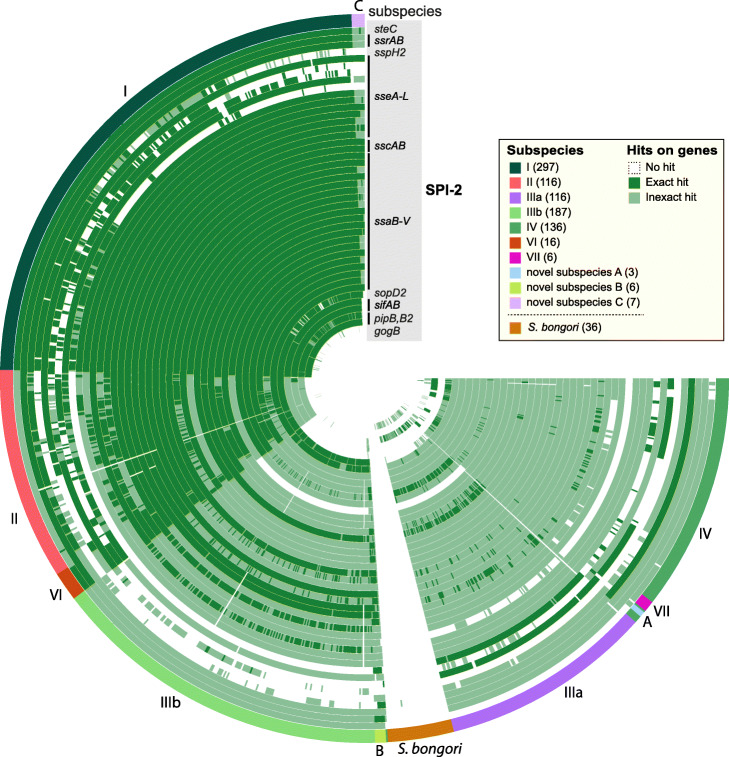
BlastFrost presence/absence analysis for 44 genes of the *Salmonella* SPI-2 pathogenicity island among 926 representative *Salmonella genomes* [20]. The outermost circle consists of one segment for each genome from *Salmonella bongori* and multiple subspecies of *Salmonella enterica*, color coded by species/subspecies (key; external text). The internal concentric circles correspond to each of the genes within SPI-2 (figure legend). Dark green: hits identified by both exact (*d*=0) and inexact (*d*=2) searches; light green: hits identified only by the inexact search; white: no hits. Graphical representation was done with Anvi’o [41]

The 44 genes in SPI-2 from *S. enterica* serovar Typhimurium strain LT2 were queried against the Bifrost graph of the 926 representative *Salmonella* genomes described above. Figure 5 shows their distribution according to an exact search (BlastFrost parameter *d*=0, dark green) and an inexact search (BlastFrost parameter *d*=2, light green). The inexact search indicated that most of the SPI-2 genes are present in all of the *Salmonella* subspecies and that they were all absent, as expected [30], in *Salmonella bongori*. However, some genes were absent from many or most of the genomes from individual subspecies, or their sequences were too divergent to be detected. This figure also emphasizes the importance of inexact querying because although most SPI-2 genes were identified by the exact search in subspecies I, II, and VI, the inexact search greatly increased the number of SPI-2 genes identified in the other subspecies.

BlastFrost took 111 s to load the Bifrost graph of 926 *Salmonella* genomes into memory and took a further 540 s to search for all SPI-2 genes with the inexact BlastFrost search, for a total of under 11 min.

#### Nucleotide variants

The subgraph extraction functionality of BlastFrost can also extract known variants of genes involved in antimicrobial resistance or other phenotypes. We downloaded 160,000 *Salmonella* draft assemblies from EnteroBase and created an initial Bifrost graph of those genomes. This took 4 days and 15 h computation time and required 147 GB of memory. During the course of the investigations in this manuscript, we subsequently updated the Bifrost graph in several iterations, resulting in a final graph containing 190,209 genomes. Updating the Bifrost graph update with 100 additional genomes took about 2.5 h, including the time to load the graph back into memory. The disk size of the final graph of 190,209 genomes is 158.5 GB and it contains 32,692,889 unitigs. We then queried this graph with BlastFrost for a single representative gene sequence from each of the genes *gyrA*, *gyrB*, and *parE*. These genes were chosen because they possess quinolone resistance-determining regions (QRDR) in which individual nucleotide variants can cause reduced susceptibility to fluoroquinolones [27]. The queries resulted in one subgraph per gene, whose sequences were aligned, and scanned for the known nucleotide variants.

**Fig. 6:**
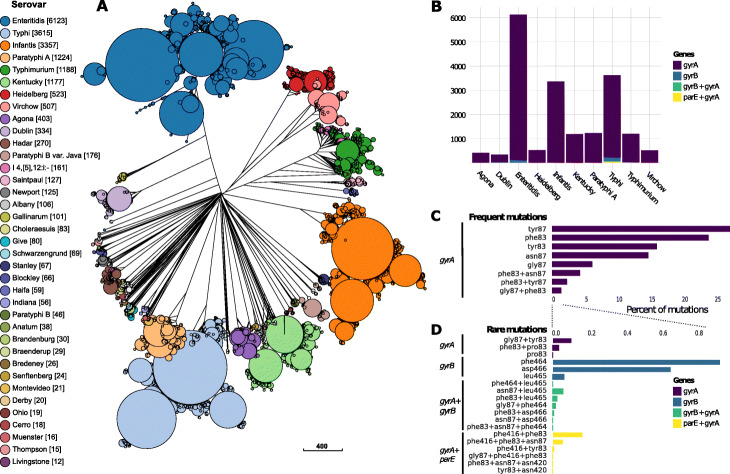
Genetic and serovar associations of 20,490 *Salmonella* genomes containing QRDR variants of genes *gyrA*, *gyrB*, and *parE* that are associated with fluoroquinolone resistance [27]. **a** Neighbor-joining tree based on cgMLST distances (visualized in GrapeTree [5]), colored by the *Salmonella* serovar in EnteroBase [3] based on SISTR1 [42] analyses of draft genomes. **b** Distribution of mutated genes by serovar for the ten most frequent serovars in **a**. **c**, **d** Percentage of individual nucleotide mutations, and their combinations for frequent (**c**) and for rare (**d**) mutations

Our results showed that 20,490 genomes from multiple serovars (Fig. 6a) contained these QRDR nucleotide variants. Those serovars included common causes of human disease, such as Enteritidis, Typhi, and Typhimurium, as well as multiple others that are common in domesticated animals but can cause food-borne gastroenteritis in humans (Fig. 5b). Most of the genomes identified in these BlastFrost queries contain a single nucleotide variant in *gyrA* (89.7%) (Fig. 6c). Variants in *gyrB* (1.8%) and *parE* (0.26%) were also found but they were less common and were normally present together with *gyrA* mutations in the same genomes (Fig. 6c,d).

**Fig. 7:**
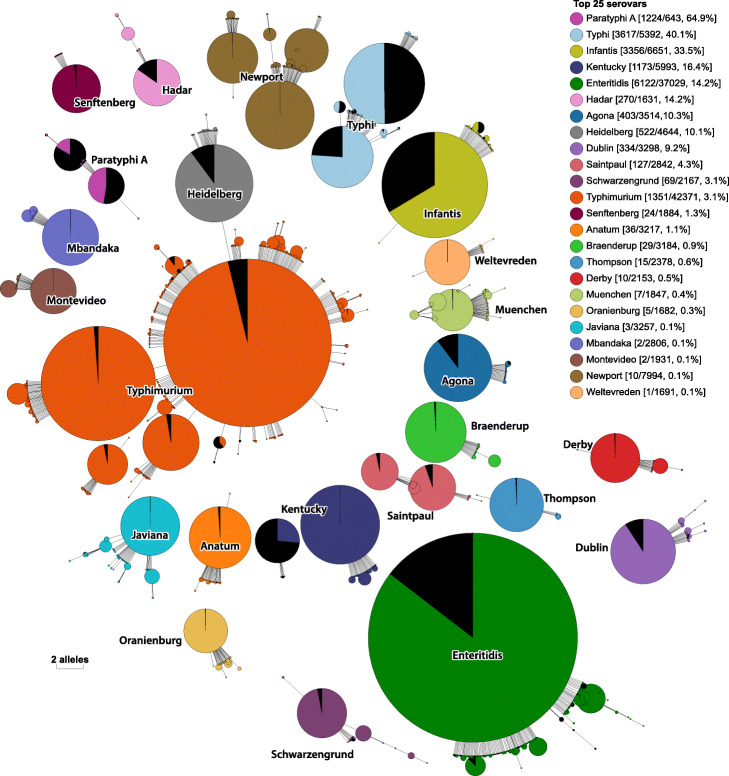
Proportion of *Salmonella* genomes containing QRDR mutations (black) among all genomes in the 25 most frequent HC900 clusters (colored) in EnteroBase [3]. HC900 clusters consist of hierarchical single-linkage clusters with maximal internal cgMLST distances of 900 alleles, which correlate well with *Salmonella* serovars. Nodes in the figure are colored and labeled by their majority serovar https:// enterobase.readthedocs.io/en/latest/HierCC_lookup.html). The neighbor-joining tree of all genomes in these clusters is based on 7-gene MLST distances and was visualized in GrapeTree [5]. For each cluster, the figure legend indicates the number of genomes with and without the QRDR mutation, and its percentage

Most of the *Salmonella* genomes from EnteroBase did not contain these QRDR mutations. The relative proportions of genomes with and without those QRDR mutations are illustrated for common serovars in Fig. 7. Serovars Paratyphi A or Typhi showed the greatest proportions of strains with resistance mutations. Interestingly, almost all fluoroquinolone-resistant strains of serovar Kentucky belong to only one of the two genetic clusters that are associated with this polyphyletic serovar [20, 32].

BlastFrost took 25 min to load the Bifrost graph into memory and 3.5 h to extract all subgraphs using 8 threads. It used a maximum of 160 GB RAM for these analyses.

## Discussion and conclusions

BlastFrost implements a highly efficient algorithm for querying de Bruijn graphs, and thereby complements the very computationally efficient Bifrost [18], which calculates compacted and colored graphs that scales to 100,000s of closely related bacterial genomes. Practical applications of the combination of these two methods are also greatly facilitated by the existence of structured sequence databases of closely related bacteria such as EnteroBase [3]. EnteroBase includes genomic assemblies of 100,000s of bacterial strains together with genotypes based on legacy or core genome MLST, which facilitate the visualization of genetic relationships among the query hits. The combination of Bifrost, BlastFrost, and EnteroBase has the potential to rapidly reveal numerous features of genomic diversity that were previously not readily accessible.

All MLST schemes are inherently limited, because they are based on a fixed selection of genes that were present in an initial, representative set of genomes. However, many bacterial genera are associated with open pan-genomes [33], whose content continues to increase with each additional genome that is sequenced [21], and such novel sequences are not routinely appended to the MLST schemes. Therefore, it is important to emphasize that BlastFrost and Bifrost are not dependent on MLST or on genomic annotations, but can handle any collection of closely related genomic assemblies. BlastFrost can summarize diversity within large, variable regions such as genomic islands. It can also identify variants of any sequence of interest and subsequently rapidly analyze them to identify single nucleotide polymorphisms.

We note that in theory, we can only guarantee that all query *k*-mers found in the graph for a specific color are really present in the underlying genome. If BlastFrost is used to query sequences containing repeats longer than the value of *k*, this could lead to false sequences returned due to the underlying data structure used. Hence, *k* should be chosen large enough to avoid false-positive sequences [34].

We compared the speed and memory requirements for large genomic data sets between BlastFrost/Bifrost, Megablast, classical BLASTN, and the current state of the art tools BIGSI and minimap2. Computing a Bifrost graph is a costly indexing step that runs much slower than creating a BLAST database. However, BlastFrost can run queries much more quickly than BLAST, which compensates for the extra time needed to create an index if that index is used repeatedly for a large number of queries. As a use case, BlastFrost would be suitable as a web service in support of large databases such as EnteroBase, because it would provide fast search functionalities and could even support comparisons of all genes from numerous whole genomes against a large pan-genome. BlastFrost is not suitable for indexing and querying diverse sequence collections such as RefSeq or SRA, unlike either BIGSI or BLAST. However, for closely related genomes, such as those within a single bacterial genus, BlastFrost is considerably faster than BIGSI and requires less memory for up to 1400 sequence queries. Similarly, BlastFrost is much faster than minimap2 for closely related genomes and also requires less memory. These computational efficiencies did not sacrifice accuracy. BlastFrost has high precision and sensitivity for sequences that are at least 90% identical and over 200 bp in length.

BlastFrost enables the identification of genomic islands or individual nucleotides associated with antimicrobial resistance genes because of the explicit graph data structure in Bifrost which supports graph traversal and extraction of sequences that extend beyond the *k*-mers that were used for querying. Given a Bifrost graph, genomic islands or nucleotide variants can be identified within 100,000s of genomes in minutes. The Bifrost API freely supports annotation of nodes in the graph, including annotating unitigs with additional data. In future extensions, BlastFrost should be able to extract the local synteny from graphs whose unitigs are annotated with genome coordinates and/or gene annotations. Such information could also be used to reconstruct genomic rearrangements.

BlastFrost is not a general replacement for calling SNPs because its precision suffers with increasing genetic diversity and reduced sequence length. However, it might have the potential for incorporation into approaches to detection of antimicrobial resistance in combinations of databases of AMR genes such as CARD [35] or AMRfinder [36] with genomic sequence collections such as EnteroBase.

## Supplementary Information

**Additional file 1** Supplementary file. Description of data structures and algorithm pseudocode

**Additional file 2** Review history

## Data Availability

The source code of BlastFrost is available as open source software at https://github.com/nluhmann/BlastFrost[37]. The source code is released under a GPL-3.0 License. The exact version used in this paper is archived at Zenodo [38]. All genome assemblies used in the analyses in this paper were downloaded from EnteroBase. For each dataset, a file containing all EnteroBase barcodes for these assemblies has been stored under https://github.com/nluhmann/BlastFrost/data [39]. The wgMLST scheme for *Salmonella* in EnteroBase can be downloaded from https://enterobase.warwick.ac.uk/species/senterica/download_data [3]. The sequences for SPI-2 genes were downloaded from the Virulence Factor Database at http://www.mgc.ac.cn/cgi-bin/VFs/genus.cgi?Genus=Salmonella[40].
