# Supplementary material for: BlastFrost: fast querying of 100,000s of bacterial genomes in Bifrost graphs
Source: Genome Biol. 2021 Jan 11;22:30. doi: 10.1186/s13059-020-02237-3 (PMC7798312; doi:10.1186/s13059-020-02237-3)
Supplement: Supplementary file 2 — Additional file 2 Review history [file 13059_2020_2237_MOESM2_ESM.docx]

Review History

**First round of review**

**Reviewer 1**

**Are you able to assess all statistics in the manuscript, including the appropriateness of statistical tests used?**

No

**Comments to author:**

The authors describe a novel tool, BlastFrost, for querying sequences in large pangenomes of bacteria. The most impressive feat here is the dynamic construction of a pangenome graph of 190k salmonella genomes. The other application shown in the manuscript (genomic islands) is significantly smaller-scale (a database of 946 genomes) and could have likely been done with existing tools. In terms of comparing the software with the state of the art, I am afraid that the manuscript misses its mark by having an unconvincing comparison with minimap2 (see my major remark below) and not attempting any comparison of BLAST nor any of its variants (e.g. Vsearch) on the smaller database, to establish that the tool can indeed act as an alternative to BLAST.

I could follow the structure of the manuscript but, going into the details, the methodological section of the main text has numerous issues (detailed below). In terms of the Results, many applications are revolving around Enterobase. It seems that Enterobase already has a 'locus search' functionality, possibly based on BLAST. I am sure that BlastFrost is more resource efficient, but the authors should at least discuss this existing functionality.

Major remarks:

1. In the Introduction, quoting: "However, these alignment-based methods do not scale well."

Further arguments should be developed for the sake of drawing a line where previous methods fail, and where Blastfrost is useful. BLAST has indexed 'nt', which is a rather large collection of genomes. According to https://www.biostars.org/p/260654/, it contains 148 Gbp. Could you be more precise regarding the expectancy of "scaling well"?

2. Figure 3C: is indexing time included in the minimap2 running time? If so, the comparison would not be fair as BlastFrost also has (non-included) indexing time.

3. I would have liked more methodological details on the computation of the E-value.

4. I would also have liked to see, even qualitatively, a comparison with BLAST. Given the name, one could believe that BlastFrost could be a substitute to BLAST, therefore how should one set expectations regarding potential similarity or dissimilarity of results, in particular with respect to lower-identity hits (sensitivity)?

5. In the Results, quoting: the paragraph that starts with "For each query, BlastFrost calculates [..]" has numerous issues. For example, "the presence or absence of sequences corresponding to that query" refers to which sequences? And, what does it mean precisely for a sequence to be "absent or present" in a color? Overall, the rest of that paragraph contains many vague statements (e.g. 'functions that are integrated in the Bifrost API' appears to be an implementation detail, 'compacted' is undefined, the 'binary sequence' definition is confusing). The whole paragraph needs to be extensively revised.

6. Figure 1:

* What is path completion? What is the greedy graph path completion procedure? This is not described anywhere in the text.

* How are the paths precisely defined? Are they all (longest) paths in an extracted subgraph? Since this is an output of the program, it seems necessary to have a precise idea of it in the main text.

Minor remarks:

1. In the Introduction, quoting: "[..] shared k-mers are automatically collapsed"

What is the meaning of 'collapsed' here? De-duplicated? But note that saying 'de-duplicated' might also imply that, by deleting k-mers, one loses track of which genome(s) a k-mer belongs to.

2. In the Introduction, quoting: "[..] for a certain k consisting [..]" : for a certain k value

3. The term "graph genomes" is ambiguous, it could mean the genomes present in the graph or the genomes displayed as (separate) graphs.

4. In the Introduction, quoting: "a link to a FASTA file" could be replaced by just "a FASTA file".

5. In the Introduction, quoting: "a set of overlapping k-mers whose size corresponds to the value of k that was used to build the Bifrost graph" : Needs to be reformatting to avoid thinking that the set has size k.

6. In the Results, quoting: "A single nucleotide substitution between a query and a color will result in k k-mers that are missed [..]". Not always? A k-mer may happen to be present in that gap due to its presence elsewhere in a genome, just by chance.

7. In the Results "unitigs" is undefined on page 6.

8. In the Results, quoting: "[..] querying all 21, 065 orthologs in the whole genome MLST (wgMLST) scheme in EnteroBase [2, 30] which were derived from a pan-genome from 537 representative genomes of the genus Salmonella."

This sentence is difficult to parse: "21065 orthologs [..] in wgMLST [..] in Enterobase [..] from a pangenome [..] from 537 genomes [..]", is this a description of the query only or is it mixed with a description of the database?

9. In the sentence "Precision and sensitivity of identifying all query variants in a pangenome", why not compare with BLAST or a more recent variant of BLAST?

10. Figure 4 should either use the term 'imperfect' or 'inexact' but not both.

11. After installing BiFrost using Conda, I tried to compile BlastFrost but it gave me the following error: "/tmp/BlastFrost/src/BlastFrost.cpp:11:10: fatal error: bifrost/ColoredCDBG.hpp: No such file or directory". I believe maybe BiFrost therefore needs to be installed from source and not through bioconda, or if it is installed through bioconda, additional paths need to be properly set to guide the compiler. Also, the readme of BiFrost has a typo: 'conda -c bioconda bifrost' should have 'install'. After compiling and installing BiFrost, I still ended up with the error "./BlastFrost: error while loading shared libraries: libbifrost.so: cannot open shared object file: No such file or directory", that I solved with " ln -s /usr/local/lib/libbifrost.* .". Finally, I ran the software using the example 'command' file in the 'testdata' but it gave me the following error: "### Number of sequences that will not be queried due to length smaller than k: 0. Error deleting file.: No such file or directory".

**Reviewer 2**

**Are you able to assess all statistics in the manuscript, including the appropriateness of statistical tests used?**

Yes: Not much statistics in this manuscript - would be good to more explicitly define how the p-values are computed.

**Comments to author:**

Luhmann et al. present BlastFrost, a new method for rapid sequence search in databases that contain many homologous sequences. The manuscript is well written and the method is clever; it represents a significant advance over the previous state of the art. In general I am very positive about this manuscript; I have, however, a few remarks.

- For the first validation experiment, it is not entirely clear to me what exactly the definition of a "successful recovery" is - the authors write: "For each locus, a[n] allelic variant was scored as successfully recovered if at least 95% of the allelic sequence stored in EnteroBase was present within the BlastFrost output.". I understand that this experiment is based on searching 926 genomes for 21065 sequences. Let's say we have a genome x and a search sequence y, and BlastFrost returns sequence z. Is it so that EnteroBase contains an allelic value for the locus that corresponds to search sequence y, and that this allelic value corresponds to a sequence y'? Is the definition of "successful recovery" that the pairwise alignment identity between y' and z is >= 0.95%? And which scoring scheme is used for computing pairwise alignments? Or is y == y'? Along similar lines, the definition of "precision" is also not entirely clear to me -- i.e. how is a false-positive hit defined?

- I don't fully understand the x axis of Figure 2, and this may be related to my previous point. The authors write: "Figure 2 depicts the sensitivity and precision of this evaluation by sequence length for pairs of queried and recovered alleles according to their average sequence identity." I think a more natural thing to plot on the x axis would be the alignment identity between queried allele and allele present in the genome, or is ("allele present in the genome" == "recovered allele"? But I don't think the later equality would always hold, because assumedly there can be multiple hits for a given query sequence? Perhaps my issues in understanding what exactly is going on is due to a lack of understanding of the structure of EnteroBase on my part, and I feel it may be helpful to elaborate on this a little bit.

- I understand that the scalability of BlastFROST is one of its key selling points, but at least for the first experiment it should be possible to do some benchmarking in terms of sensitivity and precision against other tools, like BIGSI or Mantis. For comparative purposes, it would be good to go relatively close to the maximum number of genomes that can be reasonably indexed with competing approaches (e.g. BIGSI, perhaps Mantis; from Figure 7, it seems that indexing 7000 genomes would take about a day with BIGSI), and do a sensitivity / precision comparison at this point. Similarly, the authors report minimap2 computational performance, but no accuracy figures for minimap2?

- It would also be good to better understand the impact of the k-mer neighborhood parameter d in an experiment in which "truth" is clearly defined, e.g. in the first experiment. The authors investigate the impact of d in the "Genomic islands" experiment, which is interesting, but doesn't provide a clear measure of d's impact of sensitivity / precision. Incidentally, it would also seem that higher d values could lead to more false-positive hits - is this correct?

- The experiment with 190,209 salmonella genomes is impressive. The authors write "During the course of these investigations, we updated this graph in several iterations, resulting in a final graph containing" -- why and how exactly was the graph updated? For the QRDR evaluation, the authors assumedly generated pairwise alignments between the returned search result sequences and corresponding reference sequences?

- What's the exact relationship between the BLAST-like p value and subgraph extraction? Do I correctly interpret Figure 1 in that queries that result in matches with p > 0.01 are counted as "no hits"? Also, to what extent do the authors users to make use of the computed p-values? If the authors expect users to make use of the p-values, it may not be a bad idea to show that the computed p-values are reasonably well-calibrated and distinguish between true-positive and accidental hits. In any case, it would be good to explicitly specify the formula used for computation of p-values. Also, the authors use a relatively simple algorithm for estimating alignment identity from k-mer sharing between sequences - may it be a good idea to use a more refined approach similar to e.g. the mash distance, which takes into account the exponential nature of the relationship between k-mer sharing and alignment identity?

- What happens if the query or target sequences in the graph contain repeats? I also don't fully understand the relationship between k-mer colors and unitig colors during the greedy extension step. The way I understand BiFROST graphs, "color" is a property of individual k-mers, and color patterncs can differ between k-mers that are part of the same unitig. But the authors write in the Supplement: "In order to reduce computation steps, we assume two query k-mers located on the same unitig to be labeled with the same set of colors", and later (Algorithm 1) use a "getUnitigColors()" function. It would be good to clarify this a bit.

- In Figure 3A, it seems that BIGSI_t70 exhibits super-linear runtime increases - is this correct? If this is the case, wouldn't one be better off by simply running BIGSI queries one at a time?

**Authors Response**

**Point-by-point responses to the reviewers’ comments:**

*We thank both reviewers for their detailed input and comments to our manuscript.*

*Addressing the central points of the reviewers, we have extended our paper by adding extensive comparisons of our method to BLAST (megablast). We also revised the methods section in the main text to be easier to read and parse. Please find additional direct responses to the reviewers remarks below.*

Reviewer #1: The authors describe a novel tool, BlastFrost, for querying sequences in large pangenomes of bacteria. The most impressive feat here is the dynamic construction of a pangenome graph of 190k salmonella genomes. The other application shown in the manuscript (genomic islands) is significantly smaller-scale (a database of 946 genomes) and could have likely been done with existing tools. In terms of comparing the software with the state of the art, I am afraid that the manuscript misses its mark by having an unconvincing comparison with minimap2 (see my major remark below) and not attempting any comparison of BLAST nor any of its variants (e.g. Vsearch) on the smaller database, to establish that the tool can indeed act as an alternative to BLAST.

I could follow the structure of the manuscript but, going into the details, the methodological section of the main text has numerous issues (detailed below). In terms of the Results, many applications are revolving around Enterobase. It seems that Enterobase already has a 'locus search' functionality, possibly based on BLAST. I am sure that BlastFrost is more resource efficient, but the authors should at least discuss this existing functionality.

*We thank the reviewer for the detailed comments.*

Major remarks:

1. In the Introduction, quoting: "However, these alignment-based methods do not scale well."

Further arguments should be developed for the sake of drawing a line where previous methods fail, and where Blastfrost is useful. BLAST has indexed 'nt', which is a rather large collection of genomes. According to https://www.biostars.org/p/260654/, it contains 148 Gbp. Could you be more precise regarding the expectancy of "scaling well"?

*We added additional experiments comparing BlastFrost to Blast (megablast) in order to show use cases when k-mer based methods scale better. We added Figure 3 and text in both results and discussion sections.*

2. Figure 3C: is indexing time included in the minimap2 running time? If so, the comparison would not be fair as BlastFrost also has (non-included) indexing time.

*The indexing time is not included in the running time, the index has been saved to disk before the alignment step. We added this to the text in order to avoid confusion.*

3. I would have liked more methodological details on the computation of the E-value.

*We added the reference to the BLAST approach to compute the E-value based on sequence similarity, which we adopted here.*

4. I would also have liked to see, even qualitatively, a comparison with BLAST. Given the name, one could believe that BlastFrost could be a substitute to BLAST, therefore how should one set expectations regarding potential similarity or dissimilarity of results, in particular with respect to lower-identity hits (sensitivity)?

*We added extensive comparison experiments with BLAST (megablast) on different size databases and queries, showing the different use cases in which BLAST or BlastFrost would be preferable to use. In terms of lower-identity hits, our evaluation of sensitivity and specificity on the wgMLST data (which is computed with BLAST) already showed that BlastFrost is best suited for hits with over 90% identity, and would therefore be comparable with megablast. We therefore did not add an additional qualitatively comparison, but made this point clearer in our evaluation.*

5. In the Results, quoting: the paragraph that starts with "For each query, BlastFrost calculates [..]" has numerous issues. For example, "the presence or absence of sequences corresponding to that query" refers to which sequences? And, what does it mean precisely for a sequence to be "absent or present" in a color? Overall, the rest of that paragraph contains many vague statements (e.g. 'functions that are integrated in the Bifrost API' appears to be an implementation detail, 'compacted' is undefined, the 'binary sequence' definition is confusing). The whole paragraph needs to be extensively revised.

*We revised the paragraph and hope the description of the technical details of BlastFrost is now easier to parse.*

6. Figure 1:

What is path completion? What is the greedy graph path completion procedure? This is not described anywhere in the text.

How are the paths precisely defined? Are they all (longest) paths in an extracted subgraph? Since this is an output of the program, it seems necessary to have a precise idea of it in the main text.

*The path completion step is a part of the subgraph extraction functionality in BlastFrost and described in the Results section “BlastFrost subgraph extraction” as well as more formally in Appendix paragraph “Subgraph extraction”.*

Minor remarks:

1. In the Introduction, quoting: "[..] shared k-mers are automatically collapsed"

What is the meaning of 'collapsed' here? De-duplicated? But note that saying 'de-duplicated' might also imply that, by deleting k-mers, one loses track of which genome(s) a k-mer belongs to.

*The meaning of ‘collapsed’ is that the same k-mer from different genomes will be part of the same node in the de Bruijn graph. The colouring of the nodes ensures that we keep track of which genome(s) a k-mer belongs to. We revised our explanation in this paragraph.*

2. In the Introduction, quoting: "[..] for a certain k consisting [..]" : for a certain k value

3. The term "graph genomes" is ambiguous, it could mean the genomes present in the graph or the genomes displayed as (separate) graphs.

4. In the Introduction, quoting: "a link to a FASTA file" could be replaced by just "a FASTA file”.

5. In the Introduction, quoting: "a set of overlapping k-mers whose size corresponds to the value of k that was used to build the Bifrost graph" : Needs to be reformatting to avoid thinking that the set has size k.

*We have changed the wording in the manuscript regarding these remarks.*

6. In the Results, quoting: "A single nucleotide substitution between a query and a color will result in k k-mers that are missed [..]". Not always? A k-mer may happen to be present in that gap due to its presence elsewhere in a genome, just by chance.

*Indeed, however we assume in our analysis that the value of k has been chosen large enough to avoid there hits by chance.*

7. In the Results "unitigs" is undefined on page 6.

*The term unitigs is defined on page 4 as “maximal paths of multiple, non-branching nodes that are compacted into single nodes (unitigs) by collapsing the overlaps.”*

8. In the Results, quoting: "[..] querying all 21, 065 orthologs in the whole genome MLST (wgMLST) scheme in EnteroBase [2, 30] which were derived from a pan-genome from 537 representative genomes of the genus Salmonella."

This sentence is difficult to parse: "21065 orthologs [..] in wgMLST [..] in Enterobase [..] from a pangenome [..] from 537 genomes [..]", is this a description of the query only or is it mixed with a description of the database?

*We divided this long sentence into two for easier parsing.*

9. In the sentence "Precision and sensitivity of identifying all query variants in a pangenome", why not compare with BLAST or a more recent variant of BLAST?

*The wgMLST scheme in EnteroBase is derived based on BLAST.*

10. Figure 4 should either use the term 'imperfect' or 'inexact' but not both.

*We changed the Figure legend to be consistent.*

11. After installing BiFrost using Conda, I tried to compile BlastFrost but it gave me the following error: "/tmp/BlastFrost/src/BlastFrost.cpp:11:10: fatal error: bifrost/ColoredCDBG.hpp: No such file or directory". I believe maybe BiFrost therefore needs to be installed from source and not through bioconda, or if it is installed through bioconda, additional paths need to be properly set to guide the compiler. Also, the readme of BiFrost has a typo: 'conda -c bioconda bifrost' should have 'install'. After compiling and installing BiFrost, I still ended up with the error "./BlastFrost: error while loading shared libraries: libbifrost.so: cannot open shared object file: No such file or directory", that I solved with " ln -s /usr/local/lib/libbifrost.* .". Finally, I ran the software using the example 'command' file in the 'testdata' but it gave me the following error: "### Number of sequences that will not be queried due to length smaller than k: 0. Error deleting file.: No such file or directory”.

*We are sorry for the installation problems. Unfortunately, BlastFrost does not work well with Bifrost conda installations. We have added a note to the BlastFrost repository for more specific installation instructions. We also fixed the problem with the testdata. In fact, the Error was just a warning and the BlastFrost installation was running fine at this point already. We hope to provide a direct conda installation of BlastFrost soon.*

Reviewer #2: Luhmann et al. present BlastFrost, a new method for rapid sequence search in databases that contain many homologous sequences. The manuscript is well written and the method is clever; it represents a significant advance over the previous state of the art. In general I am very positive about this manuscript; I have, however, a few remarks.

*We thank the reviewer for his very thoughtful and helpful remarks on our manuscript.*

- For the first validation experiment, it is not entirely clear to me what exactly the definition of a "successful recovery" is - the authors write: "For each locus, a[n] allelic variant was scored as successfully recovered if at least 95% of the allelic sequence stored in EnteroBase was present within the BlastFrost output.". I understand that this experiment is based on searching 926 genomes for 21065 sequences. Let's say we have a genome x and a search sequence y, and BlastFrost returns sequence z. Is it so that EnteroBase contains an allelic value for the locus that corresponds to search sequence y, and that this allelic value corresponds to a sequence y'? Is the definition of "successful recovery" that the pairwise alignment identity between y' and z is >= 0.95%? And which scoring scheme is used for computing pairwise alignments? Or is y == y'? Along similar lines, the definition of "precision" is also not entirely clear to me -- i.e. how is a false-positive hit defined?

*We rewrote the respective paragraph to clear these points up. We only used one representative allele per wgMLST allele from EnteroBase to query the Bifrost graph. For each color/genome in the BlastFrost query result, we aligned the extracted allelic variant to the representative allele in order to define a “successful recovery”. We also added more precise definitions of precision and sensitivity to the main text.*

I don't fully understand the x axis of Figure 2, and this may be related to my previous point. The authors write: "Figure 2 depicts the sensitivity and precision of this evaluation by sequence length for pairs of queried and recovered alleles according to their average sequence identity." I think a more natural thing to plot on the x axis would be the alignment identity between queried allele and allele present in the genome, or is ("allele present in the genome" == "recovered allele"? But I don't think the later equality would always hold, because assumedly there can be multiple hits for a given query sequence? Perhaps my issues in understanding what exactly is going on is due to a lack of understanding of the structure of EnteroBase on my part, and I feel it may be helpful to elaborate on this a little bit.

*Indeed, the identity plotted on the x axis is the pairwise alignment identity for a recovered allelic variant to its representative allele. We changed the wording and added some details. We assume for each colour to report only one hit per query sequence.*

I understand that the scalability of BlastFROST is one of its key selling points, but at least for the first experiment it should be possible to do some benchmarking in terms of sensitivity and precision against other tools, like BIGSI or Mantis. For comparative purposes, it would be good to go relatively close to the maximum number of genomes that can be reasonably indexed with competing approaches (e.g. BIGSI, perhaps Mantis; from Figure 7, it seems that indexing 7000 genomes would take about a day with BIGSI), and do a sensitivity / precision comparison at this point.

*We state in paragraph “Speed and RAM benchmarking” that the results for BlastFrost and BIGSI are identical, so we think further sensitivity and precision experiments are not needed. Both tools rely on similar k-mer matching strategies, so we assume sensitivity and precision to be always very similar.*

- It would also be good to better understand the impact of the k-mer neighborhood parameter d in an experiment in which "truth" is clearly defined, e.g. in the first experiment. The authors investigate the impact of d in the "Genomic islands" experiment, which is interesting, but doesn't provide a clear measure of d's impact of sensitivity / precision. Incidentally, it would also seem that higher d values could lead to more false-positive hits - is this correct?

*Running BlastFrost with a d value higher than 2 is increasing the run time significantly, but will likely not add many more hits to a search result, as such hits would result in a low p-value. We normally run BlastFrost with d=2 in all our experiments. In our evaluation of precision and sensitivity, we did not see any difference for d values higher than 2, hence we only display this result for one value of d in our figure for this experiment. We chose our experiments on the genomic island in Salmonella to show the impact of the k-mer neighborhood in a well studied example, where the distribution of this specific island is considered "known" in Salmonella. We added a few more literature references to this in the manuscript. A higher value of d did not result in any hits in S. bongori, which we think is a good example of how the k-mer neighborhood does not directly lead to false-positive results.*

*Additionally, the BlastFrost output contains the specific k-mer hit patterns for each query-colour pair and also specifies hits from the k-mer neighborhood, therefore potential false-positive hits could easily be filtered/checked subsequently if one wants to explore a higher value of d.*

The experiment with 190,209 salmonella genomes is impressive. The authors write "During the course of these investigations, we updated this graph in several iterations, resulting in a final graph containing" -- why and how exactly was the graph updated? For the QRDR evaluation, the authors assumedly generated pairwise alignments between the returned search result sequences and corresponding reference sequences?

*The graph was updated as the number of genomes available in EnteroBase grew significantly during the time it took to run all our experiments. As Bifrost has the functionality to add new genomes to existing graphs, we updated our existing graph with new genomes instead of computing this large graph from scratch every time.*

*For the QRDR evaluation, we did generate pairwise alignments to determine the types of mutation.*

What's the exact relationship between the BLAST-like p value and subgraph extraction? Do I correctly interpret Figure 1 in that queries that result in matches with p > 0.01 are counted as "no hits"? Also, to what extent do the authors users to make use of the computed p-values? If the authors expect users to make use of the p-values, it may not be a bad idea to show that the computed p-values are reasonably well-calibrated and distinguish between true-positive and accidental hits. In any case, it would be good to explicitly specify the formula used for computation of p-values. Also, the authors use a relatively simple algorithm for estimating alignment identity from k-mer sharing between sequences - may it be a good idea to use a more refined approach similar to e.g. the mash distance, which takes into account the exponential nature of the relationship between k-mer sharing and alignment identity?

*Indeed, the p-value computed is used to filter out accidental hits by removing hits with p>0.01. Our precision experiment shows that this is reasonable and does neither filter out too many true-positive hits nor keeps too many wrong hits.*

*We appreciate the reviewers suggestions for improving the estimation of alignment identity and will certainly look into developing this further in future iterations of the method and software.*

What happens if the query or target sequences in the graph contain repeats? I also don't fully understand the relationship between k-mer colors and unitig colors during the greedy extension step. The way I understand BiFROST graphs, "color" is a property of individual k-mers, and color patterncs can differ between k-mers that are part of the same unitig. But the authors write in the Supplement: "In order to reduce computation steps, we assume two query k-mers located on the same unitig to be labeled with the same set of colors", and later (Algorithm 1) use a "getUnitigColors()" function. It would be good to clarify this a bit.

*The Bifrost API allows to extract the colours of a k-mer by first extracting the uniting that contains this k-mer, and then extracting the colours associated with this k-mer on this unitig. If two k-mers are located on the same unitig, in our implementation we do not extract the colours a second time but assume the k-mers to be coloured identically. We make this simplification based on the assumption that two k-mers on the same unitig would most likely be coloured differently only due to incomplete assemblies used to build the graph. If these k-mers would significantly differ in their respective genome, we assume the unitig would be split into several nodes in the graph respectively. We simplified this in the pseudocode in Algorithm 1.*

**Second round of review**

**Reviewer 1**

The authors have addressed all my remarks. The extended comparison with BLAST is much welcome, and I believe the manuscript is now in good form for publication.

I was able to test the software and it worked on my end. A very minor detail (which doesn't need a further round of revision:) I suggest that the developers create a release as per https://github.com/nluhmann/BlastFrost/issues/5 for even easier install.

**Reviewer 2**

Luhmann et al. have now submitted a revised version of the BlastFrost paper. I remain very positive about the method per se, but I continue to see a number of issues with the presented benchmarking experiments.

Regarding the main EnteroBase-based validation experiment. Fundamentally there are three sequences: the used query sequence for a gene (A); the sequence returned by BlastFrost (B); the sequence of the queried gene present in the queried genome (C). It is still not entirely clear to me which of these sequences are compared to each other when the authors stratify results by alignment identity; when benchmarking the ability of BlastFrost to detect gene presence / absence, the right metric to stratify by would be the alignment identity between A and C.

Furthermore, I would really like to see BIGSI and BLAST results in this experiment, which includes a) equivalent sensitivity and precision statistics and b) runtime / computational performance. The authors claim that they basically showed that BIGSI and BlastFrost results are identical, and that furthermore a comparison with BLAST is unnecessary because the EnteroBase reference dataset itself is based on BLAST. I can't really agree with these points. As for BIGSI, there are different variants of the algorithm with different performance metrics (exact / inexact), and it is not necessarily clear that the high degree of concordance observed between BIGSI and BlastFrost will always generalize. The EnteroBase experiment is the main experiment based on which the authors make claims about BlastFrost's sensitivity and precision; if one is to show equivalence between BIGSI and BlastFrost, it should be based on this experiment. As for BLAST, there are a myriad ways to configure BLAST (scoring schemes etc.); when calculating their accuracy metrics, the authors apply additional filtering criteria (>90% query cover of the returned sequence); and even if BLAST can be expected to show 100% sensitivity on this dataset, it would still be important to see the precision of BLAST in this setting (and its computational performance).

Does the "Precision" plot in Figure 2 really show precision (= PPV, as I would understand it), or specificity? I wonder because the legend states how the authors define false positives; for computing precision, however, it would be sufficient to define true positives (if the set of false positives is the complement of the true positives, which I would assume?).

Furthermore, the authors' definition of "true positives" makes reference to the length of pairwise alignments; it would be important to know how exactly these were computed.

In their Response Letter, the authors state that they use d = 2 for all experiments. For the computational performance comparison experiments, however, they seem to sometimes use d = 1. If d = 2 is the right way to run the algorithm, computational performance metrics should be based on this parameter as well.

There is an additional issue I didn't quite catch during the first round of reviews. Is it correct that the sequence returned by BlastFrost is not guaranteed to be present in the indexed genomes? If this is the case, it would be important to measure how often a sequence that is not present in the identified genome is returned. This is also important because Blast and Minimap2 will always only return sequences that are really present in the indexed genomes.

**Authors Response**

**Point-by-point responses to the reviewers’ comments:**

Reviewer #1: The authors have addressed all my remarks. The extended comparison with BLAST is much welcome, and I believe the manuscript is now in good form for publication.

I was able to test the software and it worked on my end. A very minor detail (which doesn't need a further round of revision:) I suggest that the developers create a release as per https://github.com/ nluhmann/BlastFrost/issues/5 for even easier install.

**Thank you, we are currently looking into creating a release and getting a bioconda package for BlastFrost for easier installation.*

Reviewer #2:

Regarding the main EnteroBase-based validation experiment. Fundamentally there are three sequences: the used query sequence for a gene (A); the sequence returned by BlastFrost (B); the sequence of the queried gene present in the queried genome (C). It is still not entirely clear to me which of these sequences are compared to each other when the authors stratify results by alignment identity; when benchmarking the ability of BlastFrost to detect gene presence / absence, the right metric to stratify by would be the alignment identity between A and C.

**In order to compute the sensitivity in our evaluation, we aligned A and C ( = the allele stored in EnteroBase). For the precision measurement, we additionally aligned A and B for alleles not stored in EnteroBase (assuming A covers at least 90% of B as a filter criterium for all evaluated methods). We clarified this point in the manuscript.*

Furthermore, I would really like to see BIGSI and BLAST results in this experiment, which includes a) equivalent sensitivity and precision statistics and b) runtime / computational performance. The authors claim that they basically showed that BIGSI and BlastFrost results are identical, and that furthermore a comparison with BLAST is unnecessary because the EnteroBase reference dataset itself is based on BLAST. I can't really agree with these points. As for BIGSI, there are different variants of the algorithm with different performance metrics (exact / inexact), and it is not necessarily clear that the high degree of concordance observed between BIGSI and BlastFrost will always generalize. The EnteroBase experiment is the main experiment based on which the authors make claims about BlastFrost's sensitivity and precision; if one is to show equivalence between BIGSI and BlastFrost, it should be based on this experiment. As for BLAST, there are a myriad ways to configure BLAST

(scoring schemes etc.); when calculating their accuracy metrics, the authors apply additional filtering criteria (>90% query cover of the returned sequence); and even if BLAST can be expected to show 100% sensitivity on this dataset, it would still be important to see the precision of BLAST in this setting (and its computational performance).

**We extended our evaluation on the EnteroBase wgMLST dataset to include both megablast and blastn (due to differing sensitivity and runtime), as well as BIGSI. The results can be seen in Figure 2.*

Does the "Precision" plot in Figure 2 really show precision (= PPV, as I would understand it), or specificity? I wonder because the legend states how the authors define false positives; for computing precision, however, it would be sufficient to define true positives (if the set of false positives is the complement of the true positives, which I would assume?).

Furthermore, the authors' definition of "true positives" makes reference to the length of pairwise alignments; it would be important to know how exactly these were computed.

**The precision plot in Figure 2 really shows precision. We plot the proportion of TP hits to all BlastFrost/Blast/BIGSI hits respectively. We removed our definition of false positives in the figure legend, and replaced it with a more intuitive definition if the computed precision and sensitivity of the evaluated methods.*

In their Response Letter, the authors state that they use d = 2 for all experiments. For the computational performance comparison experiments, however, they seem to sometimes use d = 1. If d = 2 is the right way to run the algorithm, computational performance metrics should be based on this parameter as well.

**The right way to run the algorithm depends on the data at hand. We noted d=1 if the resulting hits are identical to d=2. We made this more consistent throughout the manuscript.*

There is an additional issue I didn't quite catch during the first round of reviews. Is it correct that the sequence returned by BlastFrost is not guaranteed to be present in the indexed genomes? If this is the case, it would be important to measure how often a sequence that is not present in the identified genome is returned. This is also important because Blast and Minimap2 will always only return sequences that are really present in the indexed genomes.

**Indeed, as for all de bruijn graph based methods, in theory we can only guarantee that all query k- mers found in the graph for a specific color is present in the underlying genome. If BlastFrost ist used in subgraph extraction mode to query sequences containing repeats longer than k, they could lead to wrong sequences due to the underlying data structure used. Therefore the value of k is important for robust searches.*

*In our evaluation on the wgMLST Salmonella dataset, we confirmed each BlastFrost hit to be present in the respective genomes with blast, and did not find any sequence that is not present in the indexed genomes.*
